# The Dying Wife

**Published:** 1863-10

**Authors:** 


### THE DYING WIFE.

Lay the gem upon my bosom,
Let me feel the sweet, warm breath,
For a strange chill o'er me passes,
And I know that it is death.
I would gaze upon the treasure
Scarcely given ere I go;
Feel her rosy, dimpled fingers
Wander o'er my cheeks of snow.

I am passing through the waters,
But a blessed shore appears;
Kneel beside me, husband dearest,
Let me kiss away thy tears.
Wrestle with thy grief, my husband,
Strive from midnight until day,
It may leave an angel's blessing
When it vanisheth away.

Lay the gem upon my bosom,
'Tis not long she can be there;
See! how to my heart she nestles—
'Tis the pearl I love to wear.
If, in after-years beside thee,
Sits another in my chair,
Though her voice be sweeter music,
And her face than mine more fair;

If a cherub call thee "father!"
Far more beautiful than this:
Love my first-born, O my husband!
Turn not from the motherless.
Tell her sometimes of her mother—
You can call her by my name!
Shield her from the winds of sorrow,
If she errs, oh! gently blame!

Lead her sometimes where I'm sleeping,
I will answer if she calls,
And my breath shall stir her ringlets
When my voice in blessing falls;
Her soft, black eye will brighten,
And wonder whence it came;
In her heart, when years pass o'er her,
She will find her mother's name.

It is said that every mortal
Walks between two angels here;
One records the ill, but blots it,
If, before the midnight drear,
Man repenteth—if uncanceled,
Then he seals it for the skies;
And her right-hand angel weepeth,
*Bowing low with veiled eyes.*

I will be her right-hand angel,
Sealing up the good for Heaven:
Striving that the midnight watches
Find no misdeed unforgiven.
You will not forget me, husband,
When I'm sleeping 'neath the sod?
Oh! love the jewel given us,
As I love thee—next to God!

